# Side-effects of cidofovir in the treatment of recurrent respiratory papillomatosis

**DOI:** 10.1007/s00405-008-0658-0

**Published:** 2008-05-06

**Authors:** Ferdinand I. Broekema, Frederik G. Dikkers

**Affiliations:** Department of Otorhinolaryngology, University Medical Center Groningen, University of Groningen, P.O. Box 30001, 9700 RB Groningen, The Netherlands

**Keywords:** Recurrent respiratory papillomatosis, Larynx, Papilloma, Cidofovir, Side-effects

## Abstract

Recurrent respiratory papillomatosis (RRP) is a chronic and difficult to treat disease of the larynx. In 1998, the first article was published that described the use of the antiviral substance cidofovir to treat this disease. Although the results are promising, there remains some concern about the potential carcinogenicity of cidofovir. There is a demand for a qualitative review of the side-effects of this medicine. In this review, the side-effects of cidofovir are investigated. Special attention was given to the potential carcinogenicity of cidofovir. For this review a search is performed in PubMed and EMBASE for relevant articles in which the use of intralesional cidofovir for patients with RRP is described. Eventually, 31 articles could be included for this review. In these articles a total of 188 patients with RRP were described who underwent therapy with intralesional cidofovir. Five of these patients have developed dysplasia of the larynx during the treatment with cidofovir. This is a percentage of 2.7. This percentage is concurrent with the incidence of spontaneous malignant degeneration of RRP (2–3%). Based on this review, it can be concluded that the use of intralesional cidofovir does not increase the risk of laryngeal dysplasia. Apart from the articles that describe the intralesional administration of cidofovir, some articles have been published in which the use of intravenous cidofovir is described as a therapy for RRP. Therefore, a summary is given on the side-effects of intralesional cidofovir as well as a summary on the reported side-effects of the intravenous administration of cidofovir. Based on the outcomes of this review, recommendations are given for a safe use of cidofovir for treatment of recurrent respiratory papillomatosis in the future.

## Introduction

Recurrent respiratory papillomatosis (RRP) is a chronic disease of the larynx which is difficult to treat. The disease is caused by an infection with human papilloma viruses (HPV). In most cases, the HPV-subtypes 6 and 11 are found. Rarely the subtypes 16 and 18 are identified [48]. The symptoms of this uncommon disease may vary from hoarseness to severe obstruction of the airway. In 1998, the first article was published that described the off-label use of cidofovir to treat RRP [51].

Cidofovir is a cytosine nucleotide analogue with antiviral activity that is approved by the US Food and Drug Administration (FDA) for the treatment of cytomegalovirus (CMV)-retinitis in persons with acquired immunodeficiency syndrome. For the treatment of CMV-retinitis the cidofovir is administered intravenously in a dose of 5 mg/kg. Since 1998 several studies have been published in which the results of cidofovir in the treatment of RRP were analysed [6, 12, 20, 30, 36, 42, 43, 45, 48]. In most of these studies the therapy with intralesional cidofovir proved to be effective.

Although few side-effects of cidofovir have been reported there is some concern about the potential carcinogenicity of cidofovir. A study in rats has shown a significant increase in mammary adenocarcinomas [24]. There have also been some reports of dysplasia in humans after the use of intralesional cidofovir [20, 48, 51, 55]. However, spontaneous malignant degeneration of RRP is possible. The reported incidence of this malignant degeneration is 2–5% [29, 51]. For these percentages no distinction was made between different HPV-subtypes. Spontaneous malignant degeneration occurs in both juvenile-onset RRP and adult-onset RRP and carries a dismal prognosis [29].

Malignant transformation happens more often in patients who have a history of smoking or radiation [5]. HPV-types 16 and 18 are considered high-risk types because they are frequently associated with severe atypia and invasive carcinoma in uterine cervical epithelium [34]. Nevertheless, several articles have been published in which only the HPV-subtypes 6 and 11 were found in patients with RRP that had undergone malignant degeneration [8, 16, 27, 31, 34, 41, 44, 49]. The role of cidofovir in malignant degeneration of RRP remains unclear. Because of the increasing use of cidofovir for RRP, there is a demand for a qualitative review of the side-effects of this drug.

In this review, a summary is presented about the reported side-effects of cidofovir. Furthermore, the reported cases of dysplasia in humans after the use of intralesional cidofovir are compared to the total number of patients treated with cidofovir that have been reported in the literature.

## Materials and methods

A search was performed for articles that describe the use of intralesional cidofovir in patients with RRP. The first search was performed in PubMed (1966 to August 2007):

1. Cidofovir (laryngeal OR respiratory) AND papillomatosis
2. Cidofovir [Substance Name]
3. Papilloma [Mesh]
4. (“cidofovir” [Substance Name]) AND (“Papilloma”[Mesh])
5. #1 OR #4

Sixty-three articles were found. The following inclusion criteria were applied:

- use of intralesional cidofovir as a primary or adjuvant therapy in the larynx in human patients with RRP;
- description of the used dosage of cidofovir;
- description of observed side-effects;
- study designs have to be randomized controlled trials (RCTs), comparative studies, case series or case reports.

After selection with the above criteria a total of 28 articles could be included for this review.

A search in EMBASE (1966 to August 2007) was also performed:

#1. Cidofovir AND (‘laryngeal papillomatosis’ OR ‘respiratory papillomatosis’)

This way 104 articles were found from which 57 were also found in the PubMed search. The other 47 articles were screened with the above inclusion criteria. Another three articles could be included to a total of 31 articles for this review. Reference lists from relevant articles, including other reviews, were searched. No articles were excluded due to language.

The PubMed search that included 63 articles was also scanned for articles that describe other ways of application of cidofovir. Four articles were found that describe the intravenous use of cidofovir. In one article the cidofovir was nebulized.

## Results

Thirty-one original articles met the original inclusion criteria. They comprised 1 case-control series, 26 case-series, 4 case-reports, and no randomized controlled trials. A summary of the articles is presented in Table 1.

**Table 1:** Summary of reviewed studies

Reference	Design	No. of patients	Mean age at start cidodovir (range)	Adults (A)/children (C)^a^	Type adult/juvenile papillomatosis^b^	Gender male (M)/female (F)
Pudszuhn et al. [48]	Case series	10	45 (2–74)	8 A/2 C	7 AO/3 JO	5 M/5 F
Naiman et al. [36]	Case series	16	12 (1–34)	4 A/12 C	JO	7 M/9 F
Naiman et al. [35]	Case series	19	36 (24–52)	19 A	AO	13 M/6 F
Chung et al. [12]	Case series	11	7 (N/A)	11 C	JO	7 M/4 F
Sheahan et al. [50]	Case series	4	8 (3–16)	4 C	JO	4 F
Pontes et al. [43]	Case series	10	40 (27–58)	10 A	N/A	N/A
Dikkers [20]	Case series	9	36 (22–50)	9 A	7 AO/2 JO	6 M/3 F
Wemer et al. [55]	Case report	1	28	1 A	N/A	1 F
Palomar et al. [40]	Case report	1	4	1 C	JO	1 M
de Bilderling et al. [18]	Case report	1	8	1 C	JO	1 F
Askew et al. [3]	Case series	4	10 (8–14)	4 K	JO	2 M/2 V
Lee et al. [30]	Case series	16	33 (9–68)	13 A/3 C	12 AO/4 JO	12 M/4 F
Co et al. [13]	Case series	5	36 (30–43)	5 A	AO	1 M/4 F
Mandell et al. [32]	Case-control series	7 (incl. 3 controls)	7 (1–14)	4 C	JO	1 M/3 F
Peyton et al. [42]	Case series	11	5 (3–13)	11 C	JO	N/A
Avelino et al. [4]	Case series	5	5 (3–9)	5 K	JO	3 M/2 V
*Naiman et al. *[37]	*Case series*	*26*	*28 (5–56)*	*19 A*/*7 C*	*16 AO*/*10 JO*	*15 M*/*11 F*
*Neumann et al. *[39]	*Case series*	*7*	*48 (5–70)*	*6 A*/*1 C*	*5 AO*/*2 JO*	*3 M*/*4 F*
Chhetri et al. [11]	Case series	5	5 (1–11)	5 C	JO	3 M/2 F
Milczuk [33]	Case series	4	4 (3–7)	4 C	JO	2 M/2 F
Pransky et al. [45]	Case series	11	5 (2–9)	11 C	JO	7 M/4 F
*Akst et al. *[1]	*Case series*	*11 *	*7 (2–14)*	*11 C*	*JO*	*7 M*/*4 F*
*Coulombeau et al. *[14]	*Case series*	*26*	*28 (5–56)*	*19 A*/*7 C*	*16 AO*/*10 JO*	*15 M*/*11 F*
El Hakim et al. [22]	Case series	2	4 (3–4)	2 C	JO	1 M/1 F
Bielamowicz et al. [6]	Case series	13	47 (18–85)	13 A	N/A	9 M/4 F
Chhetri et al. [9]	Case series	5	44 (21–62)	5 A	N/A	5 M
El Aatmani et al. [21]	Case report	1	10	1 K	JO	1 V
*Pransky et al. *[46]	*Case series*	*10*	*5 (2–9)*	*10 C*	*JO*	*8 M*/*2 F*
Wilson et al. [56]	Case series	3	41 (30–63)	3 A	AO	3 M
*Pransky et al. *[47]	*Case series*	*5*	*3 (2–5)*	*5 C*	*JO*	*4 M*/*1 F*
Snoeck et al. [51]	Case series	17	44 (11–77)	14 A/3 C	N/A	10 M/7 F
Total		188	26 (1–85)	104 A/84 C	53 AO/89 JO/46/N/A	98 M/69 F/21 N/A

Six articles are shown in italics because of an overlap in patient population. The data of the patients from these articles are not added to the total number of patients

*N/A* not available

^a^Adults (above 18 years of age), children (under 18 years of age)

^b^Adult-onset (*AO* start of RRP above 18 years of age), Juvenile-onset (*JO* start of RRP under 18 years of age)

Six of the 31 included articles proved to have an overlap in patient population with one of the other articles. When corrected for this overlap a total of 188 patients remained. The mean age of these patients is 26 years with a range of 1–85 years.

The patient population consisted of 98 males (52%) and 69 females (37%). In 21 patients (11%) the gender was not reported. Of this population, 104 patients (55%) were adults (above 18 years of age) and 84 patients (45%) were children (under 18 years of age). Adult-onset RRP was diagnosed in 53 patients (28%) and 89 patients (47%) were diagnosed with juvenile-onset RRP. In 46 patients (25%) it was not noted at what age the disease started.

### Side-effects of cidofovir

The included articles have reported few side-effects. In the report of Snoeck et al. [51], two patients developed an immediate cutaneous rash after cidofovir injection on one occasion. It is unclear if the rash was a direct side-effect of the cidofovir. Such rashes are also known to happen to patients under total intravenous anaesthesia. Rashes were not further observed during the following sessions of treatment of these two patients.

In the same report one patient experienced a headache after each injection, which responded to symptomatic therapy. Another patient reported precordialgia on the days following each of his three injections. This patient was known for coronary insufficiency, but his electrocardiogram (ECG) did not show any changes. Inflammation, scarring or fibrosis have not been observed in any of the patients in this report.

Bielamowicz et al. [6] reported a local inflammatory response after injection with cidofovir. In this study, 14 patients received cidofovir in a concentration of 4.17–6.25 mg/ml. The inflammatory response was frequently observed during the 7th to 14th-day period after each injection. No new areas of scarring, web formation, or impaired vibration of the vocal fold mucosa were identified in this study.

In a report by Lee et al. [30] in which 13 patients were treated with cidofovir, 3 patients were found to have significant vocal fold scarring. In addition, one patient developed a supraglottic web after bilateral false vocal fold injections. The authors note that it is hard to say if the vocal fold scarring in this study should be attributed to the cidofovir or the repeated microlaryngoscopy.

One case was reported in which a compromised airway following treatment with cidofovir occurred [18]. An 8-year-old female patient developed glottis oedema after intralesional injection with cidofovir. This led to severe postoperative stenosis of the airway requiring intubation and 24 h mechanical ventilation.

One article was published in which the RRP was treated with percutaneous injection of cidofovir under local anaesthesia [9]. All patients in this study reported a mild stinging sensation as the medication was administered. There were no other complications related to the injection.

In the studies that monitored blood chemistry, no effect was noted of intralesionally injected cidofovir on haematological and chemical parameters in blood [33, 37, 38, 45, 51]. In two studies, the plasma cidofovir concentration after intralesional administration was measured [38, 51]. The used concentration of cidofovir in the first study was 2.5 mg/ml. The measured concentrations of cidofovir in plasma ranged from 0.36 to 0.6 μg/ml [51]. In the second study, a concentration of cidofovir of 7.5 mg/ml was used. The measured plasma cidofovir concentrations in this study ranged from 0.04 to 3.69 μg/ml [38]. In this last report, a linear relationship was found between plasma concentration and dose of cidofovir in children. In adults, this relationship was not found: they showed a greater diffusion than children with great individual variation. Therefore, the authors of this last report recommend that intralesional cidofovir should be used at a dose below that reported to lead to toxicity in IV use (3 mg/kg) [54].

The measured plasma cidofovir concentrations in the two above studies are remarkably lower than those achieved with systemic administration of cidofovir. In a pharmacokinetic study peak serum cidofovir concentrations of 7 and 24 μg/ml were found after cidofovir infusions of, respectively, 3 and 10 mg/kg [15].

### Carcinogenicity of cidofovir

There have been some reports of dysplasia in humans after the use of intralesional cidofovir [20, 48, 51, 55]. To assess the potential carcinogenicity of cidofovir, a summary of the used dosage and concentration of cidofovir and reported dysplasia in the different articles is presented in Table 2.

**Table 2:** Summary of used cidofovir and reported dysplasia in reviewed studies

Reference	No. of patients	Cidofovir (mean value, range in parentheses)						Dysplasia
		No. of injections	Concentration (mg/ml)	Cumulative dose (mg)	Injection interval	Therapy period (months)	Follow-up period (months)	
Pudszuhn et al. and Neumann et al.^a ^[39, 48]	10	4.3 (2–7)	5	33.8 (9–80)	4 weeks	N/A (2–15)	19 (8–30)	1 patient
Naiman et al. and Coulombeau et al.^a ^[14, 36, 37]	16	9 (2–26)	5–7.5	N/A	2–4 weeks	24 (1–68)	33.6 (12–76)	None
Naiman et al. and Coulombeau et al.^a ^[14, 35, 37]	19	4.5 (1–11)	5–7.5	N/A	2–4 weeks	N/A (1–47)	24 (8–57)	
Chung et al. and Akst et al.^a, b ^[1, 12]	6	4 (N/A)	5	22.4 (7–42.5)	1 month	4 (N/A)	30.2 (10–45)	None
	5	8 (N/A)	5–10	73.8 (52–107)	1 month	8 (N/A)		
Sheahan et al. [50]	4	10.5 (8–18)	5	204.8 (N/A)	3–5 weeks	10.5 (7–16)	N/A	None
Pontes et al. [43]	10	4.2 (2–8)	6	75.6 (36–144)	N/A	N/A (6–24)	N/A	None
Dikkers [20]	9	9 (6–17)	2.5	64 (10.5–128)	6 weeks	N/A	20 (6–36)	1 patient
Wemer et al. [55]	1	N/A	5	N/A	N/A	27	N/A	1 patient
Palomar et al. [40]	1	1	2.5	N/A	N.V.T.	<1	12	None
de Bilderling et al. [18]	1	32	N/A	N/A	1.5 months	31	24	None
Askew et al. [3]	4	7 (2–15)	5	N/A	N/A	N/A	N/A	None
Lee et al. [30]	16	3.5 (1–9)	2.5–5	129 (25–360)	3 weeks	N/A	25.4 (13–48)	None
Co et al. [13]	5	4.4 (3–7)	7.5	75 (45–112.5)	1 month	3.5 (2–6)	6.5 (2–10)	None
Mandell et al. [32]	4	4 (2–7)	5	N/A	2 months	10 (4–18)	26.5 (20–30)	None
Peyton et al. [42]	11	6 (4–16)	5	N/A	2 weeks	N/A	30 (N/A)	None
Avelino et al. [4]	5	8 (5–10)	7.5	N/A	2–3 weeks	N/A	14 (12–16)	None
Chhetri et al. [11]	5	13 (8–22)	5	N/A	2 weeks	15 (8–29)	15 (N/A)	None
Milczuk [33]	4	6 (6–7)	5	52 (46–63)	6–8 weeks	9.5 (8–13)	14 (12–16)	None
Pransky et al.^a ^[45–47]	11	9 (4–28)	2.5–5	N/A	2–4 weeks	9 (2–51)	43 (9–65)	None
El Hakim et al. [22]	2	3	5	N/A	2 weeks	1	12	None
Bielamowicz et al. [6]	13	6 (1–19)	4.17–6.25	140 (N/A)	1 months	12 (1–44)	N/A	None
Chhetri et al. [9]	5	7 (2–12)	37.5	348 (N/A)	2–4 weeks	12 (7–16)	12 (7–16)	None
El Aatmani et al. [21]	1	2	15	N/A	3 months	3	N/A	None
Wilson et al. [56]	3	N/A	4.17	N/A (21–42)	2–4 weeks	N/A	24	None
Snoeck et al. [51]	17	7 (2–15)	2.5	116 (30–259)	2–4 weeks	5 (1–13)	15 (2–27)	2 patients
Total	188							5

^a^Overlap in patient population

^b^Data split because of two different treatment protocols

A form of dysplasia has been reported in five patients. These five cases will be discussed in more detail in the section below.

In the report by Pudszuhn et al. [48], a 58-year-old female patient developed dysplasia during treatment with intralesional cidofovir. This patient had histologically confirmed adult-onset papillomatosis without known radiation or tobacco abuse. After five injections with cidofovir to a total dose of 30 mg in 4 months, a histological check-up revealed severe dysplasia of the epithelium with local transition to a carcinoma in situ. Therapy with cidofovir was discontinued. She was checked microlaryngoscopically and histologically without further carcinoma findings. In the clinical check-up 19 months after diagnosis, there was no evidence of a papilloma or malignoma. Virus typing was not performed.

In the report by Dikkers [20] a case of dysplasia was also found. From the original biopsies of this 45-year-old non-smoking male patient with adult-onset papillomatosis the HPV-subtypes 6 and 11 were identified. He received a total dose of 83.5 mg cidofovir in six injections. A biopsy 6 months post-cidofovir revealed moderate epithelial dysplasia without invasive growth. He had a pre-existent anterior glottic web, which theoretically might have hampered dilution of the injected cidofovir because of the decreased local blood flow.

Wemer et al. [55] describe the case of a 28-year-old female patient who developed dysplasia during treatment with intralesional cidofovir. Her initial pathological diagnosis was papillomatosis with mild dysplasia. She had no history of irradiation or cigarette smoking. After 17 months of treatment biopsy of a papilloma resection demonstrated moderate dysplasia. After 28 months of cidofovir-therapy biopsy demonstrated both moderate and severe dysplasia within the papillomas. Use of cidofovir was discontinued at this time. Three months after discontinuation of treatment persistence of moderate and severe dysplasia was demonstrated. All lesions submitted to biopsy were genotyped and found to be HPV-6-positive.

In the report by Snoeck et al. [51], two patients are described who showed lesions microscopically compatible with verrucous carcinoma. Both were smoking, male patients: one was 66-year-old, the other 77-year-old. They had received cidofovir for, respectively, 6.5 and 4.5 months. Additional investigation, however, has revealed that the pre-treatment biopsy specimens of these two patients showed atypical cells that may have actually been verrucous carcinoma rather than RRP [47]. Late biopsies taken from the 66-year-old patient were characterized by persistent hyperkeratosis, but in situ hybridization for HPV was negative. In the 77-year-old patient HPV typification was not performed.

In five of the 188 patients in this review, moderate to severe dysplasia has been reported during or after intralesional treatment with cidofovir. This means that in 2.7% of the patients treated with cidofovir dysplasia or malignant degeneration has occurred. This percentage is concurrent with the incidence of spontaneous malignant degeneration of RRP (2–3%). The patients in whom dysplasia has occurred consist of three adult male and two adult female patients with a mean age of 55 (28–77) years. Two patients have adult-onset RRP; in three patients was not noted when the disease started. The dysplasia was observed after an average period of 8 months of cidofovir treatment with a range of 4–17 months. In two of the five patients the cumulative dose of cidofovir is known. These two patients received amounts of 30 and 83.5 mg cidofovir. The HPV-subtype could be identified in two patients: once only subtype 6 was found and once both subtypes 6 and 11 were found.

### Systemic treatment with cidofovir

Apart from the articles that describe the use of intralesional cidofovir, four articles have been published that describe the use of intravenous cidofovir as treatment for RRP [2, 17, 18, 53]. The four reported cases all had pulmonary extension of their RRP. They received the intravenous injections with cidofovir at a dose of 5 mg/kg. In all cases the intravenous injections were associated with hyperhydration and probenecid in order to diminish nephrotoxicity. From the four patients with RRP that have been treated with intravenous cidofovir one displayed side-effects. She developed leukopenia and partial alopecia on her combination therapy of cidofovir and interferon [17]. At that time she had received 34 intravenous injections with cidofovir in a period of 16 months. The therapy was well tolerated in the other three patients. They received a total of 27, 23 and 37 intravenous injections in a period of, respectively, 14, 12 and 27 months [2, 18, 53]. In the treatment of CMV-retinitis of AIDS-patients, nefrotoxicity and neutropenia have been reported as side-effects of the therapy with intravenous cidofovir [28].

### Nebulized treatment with cidofovir

Apart from the four articles in which cidofovir was administered intravenously to treat the pulmonary extension of RRP, one article is published in which a case is described where the cidofovir was nebulized to reach the pulmonary lesions [26]. In this case a girl presented at 11 months of age with respiratory failure and stridor due to her RRP for which a tracheostomy was performed. To reach the lung papillomas cidofovir was nebulized through her tracheostomy tube (20 mg/ml, 4 ml, 3 times/week). Her only complication was hemoptysis that resolved with decreasing the dosage to 10 mg/ml, 4 ml, 3 times/week. Her lower airway lesions responded and diminished in size. Currently, her lower airway lesions have visually disappeared. She is maintained on cidofovir two times a week without complications.

## Discussion

In the literature, three different ways to administer cidofovir as treatment for RRP have been described. In most cases, the cidofovir is administered intralesionally. Four cases have been described in which the cidofovir was administered intravenously and in one case the cidofovir was nebulized to treat RRP.

In the thirty-one articles that describe the use of *intralesional cidofovir* few side-effects have been reported. The only reported *side-effects* that have an evident causal relationship with the cidofovir injections are the local inflammatory responses in 14 adult patients described by Bielamowicz et al. [6]. The development of a supraglottic web in one patient and scarring of the vocal folds in three patients described by Lee et al. [30] could have been caused by the injections with cidofovir but could have also been caused by the repeated microlaryngoscopy. One case is described in which treatment with intralesional cidofovir in an 8-year-old girl led to glottis oedema that required intubation [18].

In two studies, the plasma cidofovir concentration after intralesional administration has been measured [38, 51]. The highest measured serum cidofovir concentration was 3.69 μg/ml. This concentration was found in a child who received a dose of 2.8 mg/kg. The concentration of 3.69 μg/ml is corresponding to a dose of 1 mg/kg in intravenous infusion [38]. As the used dose of intralesional cidofovir for the treatment of CMV-retinitis is 5 mg/kg, the serum cidofovir concentrations in these patients are remarkably higher [15]. In none of the reviewed studies a concentration higher than the recommended 3 mg/kg was administered in adults.

To assess the potential *carcinogenicity* of cidofovir, all the articles that describe the use of intralesional cidofovir in patients with RRP have been reviewed. This review showed that dysplasia occurred in 2.7% of all patients with RRP that have been treated with intralesional cidofovir. This percentage is concurrent with the reported incidence of spontaneous malignant degeneration of RRP (2–3%)[51]. Based on the outcomes of this review it can be concluded that the use of intralesional cidofovir does not increase the risk of laryngeal dysplasia. The two patients with verrucous carcinoma that have been described by Snoeck et al. [51] have been included in this review. However, it remains uncertain if the pre-treatment lesions of these two patients were not verrucous carcinoma rather than RRP [47]. When these two patients would be excluded from this review there would have been three patients with dysplasia on a total of 186 treated patients. This corresponds to a percentage of 1.6. Since it is not definitely sure that the verrucous carcinoma was present before the start of therapy with cidofovir the two patients have been included in our study. The follow-up period of the included studies varied. It cannot be ruled out that there are patients in which dysplasia has occurred after the follow-up period.

In two of the five patients with dysplasia the HPV-subtype has been identified. In these two patients HPV-6 and HPV-11 were found. The high-risk types HPV-16 and 18 were not present. This is not unusual, as becomes clear from the various articles that have been published in which only the HPV-subtypes 6 and 11 were found in patients with RRP that had undergone malignant degeneration [8, 16, 27, 31, 34, 41, 44, 49].

The known cumulative dosages in the patients in which dysplasia has occurred (30 and 83.5 mg) do not differ from the cumulative dosages that have been administered in other studies. In one study, the mean cumulative dose is even 348 mg [9]. A cumulative effect on the development of dysplasia is, therefore, unlikely.

The safety of intralesional injections with cidofovir has also been evaluated in animals [10, 24, 52]. Chhetri et al. [10] used a canine model to investigate the effects of cidofovir. A 6-month period of intralaryngeal injection of cidofovir induced dose-dependent localized toxic injury to muscle and induced vocal fold atrophy and scarring. These effects appeared to be irreversible in the animals that received a dose higher than 20 mg of cidofovir. There were no clinically significant changes in leukocyte count, renal parameters, electrolytes, or liver function tests. No evidence of dysplasia, metaplasia, or carcinoma of the vocal folds was observed.

Spiegel et al. [52] evaluated the local effects of cidofovir injection on cartilage in rabbits during 6 weeks. A total of 96 sites were injected with cidofovir in concentrations of 0, 5, 25, and 75 mg. One third of the injection sites showed a gross change in appearance (mild to moderate crusting, some with erythema), whereas two thirds showed no visible change at any time during the 6 weeks. Although there was a statistical likelihood for increased local change after cidofovir injection, there was no correlation of severity with injected dose.

In a 26-week intravenous toxicology study in which rats received 0.6, 3, or 15 mg/kg cidofovir once weekly, a significant increase in mammary adenocarcinomas in female rats was observed [24]. Furthermore, a significant incidence of Zymbal’s gland carcinomas in male and female rats was seen at the high dose but not at the lower two doses. Cidofovir caused no tumours in a 1-year monkey toxicity study. In this study, cynomolgus monkeys received intravenous cidofovir, alone and in conjunction with concomitant oral probenecid, intravenously once weekly for 52 weeks. They received doses resulting in exposures of approximately 0.7 times the human systemic exposure at the recommended dose of cidofovir.

The findings in rats raised the concern about the potential carcinogenicity of cidofovir. It is noteworthy, however, that the development of mammary adenocarcinoma in rats is a frequent occurrence in rat pharmacologic studies [19].

In the four cases in which the cidofovir was administered *intravenously* the RRP had an aggressive course with pulmonal extension. Pulmonary involvement of RRP can lead to respiratory insufficiency and even death [45]. Few side-effects have occurred in the four cases in which the cidofovir was administered intravenously [2, 17, 18, 53]. In one case partial alopecia and leukopenia occurred after 34 injections with cidofovir in a period of 16 months [17]. Nefrotoxicity was not noted in the four described cases. Therefore, pulmonary involvement of RRP seems a justified indication for the intravenous administration of cidofovir. The administration of oral probenecid and intravenous hydration before and after each cidofovir dose remains important.

Because there are no adequate and well-controlled studies in *pregnant women* and cidofovir has shown to be teratogenic and embryotoxic in animal studies, this medicine is classified by the FDA as a pregnancy category C drug [25]. The FDA recommends using category C drugs only during pregnancy if clearly needed [23]. Because RRP can also be controlled with repeated microsurgery the use of cidofovir during pregnancy can be avoided. It is noteworthy, however, that the recommendations of the FDA for cidofovir during pregnancy are based on the systemic administration of cidofovir.

In a combined in vitro and in vivo study on the *teratogenicity of cidofovir* a cytostatic effect on eukaryotic cells and a generalized embryolethal effect in pregnant rats was found [7]. The cidofovir was administered to the rats in a relatively high concentration of 20–100 mg/kg. For the intralesional administration of cidofovir concentrations below 3 mg/kg are being used. As noted above the plasma cidofovir concentrations after intralesional administration are remarkably lower than those achieved with the systemic administration of cidofovir [38]. Furthermore, no systemic side-effects have been reported during treatment with intralesional cidofovir. Therefore, the question rises if teratogenic effects are indeed to be expected during the use of intralesional cidofovir. More studies in the future are necessary to answer this question. Until that time it remains recommended to be reserved with the use of intralesional cidofovir during pregnancy.

## Conclusion

Based on the published literature it can be concluded that the therapy with intralesional cidofovir for patients with RRP does not increase the risk of laryngeal dysplasia. Systemic side-effects have not been reported in patients who have received intralesional cidofovir. Treatment with intralesional cidofovir seems to be safe provided that the regulations for this therapy are being followed. These regulations comprehend that the intralesionally injected dose of cidofovir in adults should be below 3 mg/kg. During pregnancy, it is recommended to be reserved with the use of intralesional cidofovir. Pulmonary involvement of RRP seems a justified indication for the intravenous administration of cidofovir.

The treatment with cidofovir should be given in a tertiary care hospital because of the low incidence of RRP. Furthermore, it remains important to monitor the potential side-effects of cidofovir and always histologically analyze the biopsies taken from patients who received treatment with cidofovir. The publication of encountered side-effects and occurrence of dysplasia during treatment with intralesionally applied cidofovir is important for the evaluation of the safety of cidofovir in the future.
